# Lifestyles and sexuality in men and women: the gender perspective in sexual medicine

**DOI:** 10.1186/s12958-019-0557-9

**Published:** 2020-02-17

**Authors:** Daniele Mollaioli, Giacomo Ciocca, Erika Limoncin, Stefania Di Sante, Giovanni Luca Gravina, Eleonora Carosa, Andrea Lenzi, Emmanuele Angelo Francesco Jannini

**Affiliations:** 1 Department of Systems Medicine, University of Rome Tor Vergata, Via Montpellier 1, 00133 Rome, Italy; 2 Department of Experimental Medicine, Section of Medical Pathophysiology, Food Science and Endocrinology, Sapienza - University of Rome, Viale Regina Elena 324, Rome, 00161 Italy; 3 Department of Biotechnological and Applied Clinical Sciences, University of L’Aquila, Via Vetoio (Coppito 2), L’Aquila, 67100 Italy

**Keywords:** Sexual dysfunction, Erectile dysfunction, Coital pain, Loss of libido, Obesity, Mediterranean diet, Chronic stress, Substance use, Alcohol dependence, Physical activity

## Abstract

Sexual health is strictly related with general health in both genders. In presence of a sexual dysfunction, the expert in sexual medicine aims to discover the specific weight of the physical and psychological factors can cause or con-cause the sexual problem. At the same time, a sexual dysfunction can represent a marker of the future development of a Non-communicable diseases (NCDss) as cardiovascular or metabolic diseases.

In the evaluation phase, the sexual health specialist must focus on these aspects, focusing especially on the risk and protective factors that could impact on both male and female sexuality.

This article presents a review of researches concerning healthy and unhealthy lifestyles and their contribute in the development of sexual quality of life in a gender-dependent manner.

Among the unhealthy lifestyle, obesity contributes mostly to the development of sexual dysfunctions, due to its negative impact on cardiovascular and metabolic function. Tobacco smoking, alcohol - substance abuse and chronic stress lead to the development of sexual dysfunction in a med-long term.

In order to guarantee a satisfying sexual quality of life, sexual health specialists have the responsibility to guide the patient through the adoption of healthy lifestyles, such as avoiding drugs, smoke and excessive alcohol, practicing a regular physical activity, following a balanced diet and use stress-management strategies, even before proposing both pharmaco- and/or psychotherapies.

## Introduction

Sexual health is characterized by a complex and multidimensional process coordinated by neurological, endocrine, and vascular systems [1]. Male and female sexual dysfunctions represent a medical and psychological problem that adversely affect not only physical health and emotional well-being: the impairment of sexual function may have a detrimental effect on self-esteem, body image, interpersonal relationships, and physical health in general, including fertility.

The modifiable risk factors for male and female sexual dysfunctions are: smoking, physical inactivity, obesity and excessive alcohol and drug consumption. Healthy lifestyle changes could be a useful strategy for decreasing the risk of erectile dysfunction (ED) and the other sexual dysfunctions.

Since males and females differ in the expression of sexual behavior and function, both from a biological and psycho-relational point of view, the endpoint of this review is to describe how healthy and unhealthy lifestyles impact differently on male and female sexuality.

The greatest limitation in this field remains the lack of study assessing the impact of lifestyle change on sexual function. On the other hand, however, none of the many therapeutic options available to date offers a complete response in patients with sexual dysfunction, and therefore, as with many other diseases, prevention remains the most effective approach to alleviating the consequences of sexual dysfunction.

## Obesity

Obesity is a dramatically serious public health problem and in many countries, it has the characteristics of a real epidemy. Information provided by epidemiological surveillance systems suggests that almost two billion adults are overweight, 500 million of whom are obese, and for the first time, the number of obese individuals in the developed countries is almost equivalent to the number of underweight, with a higher prevalence in men [2].

Together with the high prevalence, obesity negatively impacts on physical, psychic, and sexual health in general. For example, class III obesity has been shown to reduce life expectancy from 6 to 14 years [3]. Another study reveals that the life expectancy reduction in both male and female obese worsen with smoking (from 7.2 to 13.3 years in females, from 6.7 to 13.7 in males) [4].

Obesity, by itself, is an independent risk factor for cardiovascular disease but also increases the incidence of pathologies such as diabetes, dyslipidaemia, hypertension, which, like obesity, are themselves factors of risk for the development of cardiovascular disease [5]. Among psychosocial factors, obesity causes psychological distress, in particular it is a cause of social stigma and weight-related discrimination, adversely affecting the quality of life especially in women [6]. The psychopathology that most weighs on obese people is depression. It has been highlighted that among people extremely obese, the diagnosis of a mood disorder is very common [7].

From a sexual perspective, an American study on sexual dysfunctions in the obese population showed prevalence rates around 7–22% for women (coital pain, arousal problems, and sexual dissatisfaction) and 5–21% for men (ED and decrease of desire) [8].

In addition, numerous scientific evidences [9–11] have highlighted the important interconnection between obesity, sexual dysfunction and the development of future Non-Communicable Diseases (NCDs) in general and cardiovascular diseases in particular [1]. At present, the occurrence of sexual dysfunction in obese patients provides an opportunity for the in-depth clinical trials, which in most cases allow them to identify and intervene on an early stage of cardiovascular disease [12]. Unlike men, less is known about the relationship between sexual function and the amount of body fat in the female counterpart. The lack of information is due to the scarcity of the studies and mainly to the different methods used for female sexual evaluation [1].

Patients with ED generally have a body weight and a waist circumference on average higher than healthy patients. In fact, in the Health Professionals Follow-up Study [13], men with a Body Mass Index (BMI) greater than 28.7 had a risk of developing ED more than 30% of the normal (BMI < 25). Similarly, the results of the Massachussets Male Aging Study [14] and Rancho Bernardo Study [15] studies lasting 9 and 25 years respectively showed that body weight is an independent risk factor for ED with an additional risk 90% compared to controls (Odd Ratio between 1.93 and 1.96 respectively).

A wider prevalence of ED can also be highlighted in patients with metabolic syndrome that represents a constellation of risk factors for the development of diabetes, coronary heart disease and other vascular complications. For its diagnosis it is necessary to coexist at least three of the criteria coded by the Adult Treatment Panel III: waist circumference > 102 cm in humans,> 88 cm in woman; triglycerides> 150 mg / dl; cholesterol-HDL < 40 mg / dl in humans, < 50 mg / dl in woman; blood pressure > 130 / 85 mmHg; glycemia> 110 mg / dl) [16].

More and more relevant results suggest that the association between obesity and ED may be explained by endothelial dysfunction [17]. This link has been clinically proven for the first time by Kaiser, comparing men with ED without manifest manifestations or risk factors of vascular disease with a similar control group but without ED. Between the two groups of the study, there were no significant differences in carotid-medial thickness of calcific total-score of the coronary arteries, but there was, however, a significant decrease in vasodilation of the brachial artery in the ED group. These first observations suggested that ED could be considered an extremely early manifestation of vascular insufficiency.

Regarding female sexual health, recent literature data suggest that overweight and obese women have a lower sexual function than healthy women [18, 19]. It is important to point out how the increase in the severity of obesity is positively correlated with a greater impairment of the sexual quality of life, to a greater extent than that of obese men [20, 21]. In addition, among all obese women, those who report major sexual problems are women who choose bariatric surgery [20–22]. All these results suggest that women with severe obesity, especially those seeking bariatric surgery, represent a population with a high risk of developing sexual dysfunctions.

Only a few studies investigated in a specific way the sexual function in obese women: In a study by Kirchengast et al. [23] found that in postmenopausal women a high BMI was associated with reduced sexual desire. In another study, the prevalence of female sexual dysfunction among women seeking bariatric surgery was higher than in women with infertility (26%) and women with hypertension (42%), but similar to those who accessed to the sexological outpatient clinic (60%) and lower than women with urogynecological problems (64–68%) [24].

On the other hand, Esposito et al. [25] discovered a negative relationship between body weight and sexual function in 52 women with abnormal values of FSFI (score < 23), showing that obesity affects several areas of sexual function unlike healthy women with FSD: arousal, lubrication, satisfaction, and orgasm, but not desire and pain.

Data from a study based on a sample of obese women preparing for bariatric surgery [26] confirm the Esposito’s study results, except for the area of desire, which is significantly lower in the sample of obese women than in the control group. This discrepancy could be attributed to the different impacts that, in the different studied cohorts, may have the relative hyperandrogenism frequently found in female obesity versus the loss of the body image also very common in the same condition [7, 27].

Finally, overweight, obesity and a sedentary lifestyle are associated with an increased risk of post-menopausal breast cancer [28, 29], as excessive adiposity increases the concentration of sexual hormones such as estrogen and androgen [30, 31].

## Physical activity

Physical activity is one of the healthiest activities and most of all reduces the risk of chronic diseases (such as diabetes or hypertension) or sexuality [32]. In addition, some studies have shown that an active lifestyle greatly reduces the chance of having an altered blood glucose control. or to improve it if already present [33].

In subjects with sexual dysfunction and suffering from diabetes or severe obesity, physical activity is a very important protective factor: constant physical activity has been a protective effect against ED in men with diabetes [34] as well in women with sexual dysfunction [35].

From a meta-analysis it has been shown that intense and moderate physical activity is associated with a lower risk of developing ED because it increases endothelial NO production and decreases oxidative stress. In addition, exercise has proven beneficial effects on self-esteem and mental health, with a positive impact on psychological problems associated with sexual dysfunction [14, 36].

In hypertensive patients with ED, 8-week physical exercise duration of 45 to 60 min per day improved erectile function compared to controls remaining sedentary during the same period [37]. These data were confirmed by a recent study evaluating the effect of an aerobic physical activity protocol (about 150 min per week) on the quality of erectile function in middle-aged patients with ED on a vascular basis. After 3 months, patients in the intervention group showed a significant increase in the abridged International Index of Erectile Function (IIEF-5) score associated with a reduction in pro-apoptotic endothelial cells compared to controls [38].

In another study, 60 patients with ED were randomized to receive a type 5 phosphodiesterase inhibitor (PDE5i) alone or in association with regular aerobic activity (about 3 h a week). After 3 months in the intergroup there was an improvement in the total score of the IIEF of 77.8% compared to 39.3% of the control (*p* < 0.004) suggesting that lifestyle changes can significantly increase the benefits of pharmacological therapy for ED. [39]

Similar results were founded in subjects with Premature Ejaculation (PE) [40, 41]. In a recent study, it’s been demonstrated that PE symptoms tended to increase with decreasing frequency of physical activity [42]. Being the ejaculatory mechanism itself unrelated to the lifestyle, the unique logical explanation of this effect could be the very frequent comorbidity between ejaculatory and ED [43–45], where one exacerbates the other. Moreover, physical exercises improve self-esteem and body image, which are negatively influenced in subjects with PE [46].

In women, physical exercise can improve many typical symptoms of menopause, particularly mood, sleep, anxiety, depression and musculoskeletal problems [47–49]. Together with these, postmenopausal women who do regular physical activity maintain a good quality of sexual life [48].

In a longitudinal study [50], menopausal women have a better body image when they exercise a constant physical activity, improving their self-esteem, emotion expression, and maintaining an adequate BMI.

Despite the importance of sexuality and physical activity to the quality of life and health of women, studies that evaluate the relationship between these aspects are scarce in the literature.

In a study by Cabral et al., the presence of a sexual dysfunction, measured by the Female Sexual Function Index (FSFI), was found in 67% of the sample and associated with a sedentary lifestyle. Almost 4 out of 5 women who did not exercise physical activity reported sexual dysfunction (78.9%) compared to regular physical exercise (57.6%). Based on these data, the absence of physical activity increases by 2.1 times the chance of having a sexual dysfunction in menopausal women [51].

## Loss of weight and dietary factors

Weight loss, whether through lifestyle changes or through bariatric surgery, is associated with an improvement of many biological, psychological and sexual factors.

Khoo et al. compared the effects of 2-month low calorie diet on insulin sensitivity, plasma testosterone levels, erectile function and sexual desire in obese and diabetic men compared to non-diabetic men but with similar BMI and waist circumference. 10% weight loss was significantly associated with increased insulin sensitivity, plasma testosterone levels, erectile function and desire in diabetics as well as non-diabetic patients [52]. Similar results were obtained with weight loss induced by bariatric surgery as demonstrated by the increase in the quality of erectile function measured with IIEF-5 and the increase in total testosterone levels [53].

The Mediterranean Diet, that includes a high consumption of legumes, vegetables and fruits, and the limited consumption of red meat, dairy products, high-added foods and beverages is associated with a reduction in the risk of ED and other related sexual complaints in both diabetic and non-diabetic patients.

Sixty-five men with metabolic syndrome and ED have been studied; 35 of these were assigned to the diet of intervention according to the Mediterranean diet model and 30 to another diet model. Subjects in the intervention group were invited to consume at least 250–300 g (g) of fruit, 125–150 g of vegetables, and 25–50 g of nuts per day. In addition, they were encouraged to consume 400 g of whole grain a day (legumes, rice, corn and wheat) and increase the consumption of olive oil. After 2 years, men from the Mediterranean diet group had an increased IIEF score compared to men in the control group [54].

The beneficial effect of the Mediterranean diet on atherosclerosis in general and in particular on ED is mediated by multiple biological pathways, including a reduction in oxidative stress, subclinical inflammation and improved insulin sensitivity, which in turn may increase the release of NO into penile arteries.

Diabetic women who better complied to the Mediterranean diet have reported a lower BMI, a waist circumference and a waist-to-hip ratio, lower levels of depression, obesity and metabolic syndrome, a higher level of physical activity, and better profiles of glucose and lipids compared to women who did not constantly diet. In addition, adherence to the Mediterranean diet has also improved the frequency of sexual intercourse and significantly reduced the prevalence of sexual dysfunction [55].

Moreover, another study reveal that baseline C-reactive protein seem to predict ED in men but no sexual dysfunctions in women [56].

## Smoking

Smoking is a global health problem. A cigarette contains nearly forty thousand chemical compounds, of which 60 are extremely toxic [57]. Epidemiological studies show that smoking contributes to the etiology of many diseases, including respiratory, neurological and oncological circulatory disorders, due to its toxic properties [58].

One of the main effects of cigarette smoking is the decrease in vasodilatation of vascular endothelial tissues: Chronic smoking causes ED in men, while women’s role is still controversial [59]. However, it is possible to assume that the complications related to smoking and sexual dysfunction are similar in both sexes.

Both direct use and exposure to tobacco are risk factors for the development of ED. A recent meta-analysis [60] of four prospective cohort studies and four case-control studies involving over 28,000 participants showed that compared to non- smoking, the overall odd ratio of ED in cohort prospective studies was 1.51 (95% CI: 1.34–1.71) for smokers, and 1.29 (95% CI: 1.07–1.47) for the ex-smokers.

In a prospective study, Pourmand et al. have reported a beneficial effect on erectile function in men who have ceased smoking. After 1 year, the state of ED improves by 25% in ex-smokers but in none of the current smokers [61].

According to Harte and Meston, the effects of acute nicotine intake diminish 30% of genital stimulation and cause an alteration of normal sexual response [62]. Battaglia et al. report that the anti-estrogenic effects of smoking negatively impact on uterine, clitoral and labial vascularization [63]. Negative effects are also present in orgasmic function (which is delayed) and in a decrease in vaginal lubrication [63]. Therefore, it is possible to hypothesize that smoking is a risk factor in female sexual function, although the pathophysiological mechanisms of the negative symptoms of smoking on the female sexuality are still unclear.

It is important to emphasize that one of the effects of nicotine is to have a negative impact on sex hormones (androgens and estrogens) [64], and on clitoral blood flow [65]. Therefore, sexual symptoms such as hypo lubrication can be determined by the reduced flow of genital blood. This condition can also lead to a delay in orgasm and a more general decline in sexual relationships and a sense of dissatisfaction for sexual intercourse and couple relationship [10, 64, 65].

Devices proposed in the market to be able to reduce the risk of combusted toxic of cigarette smoking must be carefully evaluated to demonstrate with robust evidences their ability to positively impact on sexual and reproductive health in both genders before to be considered as real advantages and not an alibi to remain in the smoking habit [66].

## Alcohol

Alcohol consumption and sexual behavior are closely linked. Although incorrect, there is a common belief that alcohol is a powerful sexual facilitator, acting as an aphrodisiac and pushing the person towards disinhibited behaviors [67].

Indeed, although low-dose alcohol consumption produces a slight euphoria, leading some people to be more open or receptive to sexual activity, at higher doses alcohol leads to an opposite effect as it tends to attenuate the sexual response in a gender-dependent manner. Indeed, alcohol is a central nervous system depressor that slows down brain function, breathing and blood flow [68]. Consequently, the alcohol consumption, particularly in alcohol-dependent subjects, lead to the development of a sexual dysfunction, especially to ED in men and reduced vaginal lubrication in women [68]. Although almost identical in the pathophysiology, erection and lubrication exert, however, a different, gender-dependent function in coital activity. The significant reduction in erection may obstacle penetration much more than the same reduction in lubrication. For this reason, alcohol dependence impacts on sexual performance much more in male than in female [69].

Moderate consumption of alcohol can have a protective effect on ED both in the general population and in diabetes [70]. Data from a transverse study based on the assessment of the association between habitual alcohol consumption and ED in Australia revealed that the likelihood of developing ED among drinkers was lower among men who consumed between 1 and 20 standard portions per week [71]. In general, overall results indicate that the consumption of a moderate amount of alcohol per week gives a higher degree of protection [71]. The beneficial effects of alcohol on erectile function may be partly due to the long-term benefits of alcohol on the increase in NO bioavailability. These findings need confirmation in larger populations and with different ages and comorbidities.

There is no doubt that excessive use of alcohol not only has negative effects on life expectancy, but significantly reduces the quality of sexual and relational health through neuro- and vasculo-degenerative mechanisms as well as hormone-toxicity. True alcoholism ultimately has a devastating impact on both social and sexual life [72].

Most of the women who drink large quantities of alcohol report sexual dysfunctions. In a study, female patients seeking treatment for alcohol dependence syndrome reported mostly low sexual desire (55%), inability to reach orgasm (52,5%) and, when reached, unsatisfactory (50%). Compared with healthy women, alcohol-dependent women reported lower scores in all the sexual domains [73]. Low educational levels, early alcohol initiation, longer alcohol consumption and dependence from other substances seemed to be the most significant predictor of the development of sexual dysfunction [73]. Paradoxically, despite the hypoactive sexual desire disorder, both promiscuity and frequency of (unprotected) sexual activity seem increased both in chronic and acute alcoholic abuse [74].

## Substance use

The psychological and physiological effects that illegal psychoactive substances induce are extremely variable in nature, as they depend not only on the pharmacodynamic aspect of the molecule itself but also on the mode of intake (smoking, orally, intranasally, parenterally), dose, emotional states, expectations related to the substance’s intake and the value it has accredited.

Despite the effect of drugs and alcohol on an individual’s sexual behaviors [75], sexual dysfunctions have received limited attention in the addiction field [76]. This is particularly strange, considering that the use of illegal drugs is very much related to the attempt to overcome a real of hypothetical sexual dysfunction [77].

Recently, an increasing number of people voluntary intake psychoactive drugs (*Gamma-hydroxybutyrate, gamma-butyrolactone, 1,4-butanediol, mephedrone, methamphetamine, ED drugs and alkyl nitrites*) in order to enhance sexual performance. This phenomenon, called “ChemSex”, is frequent in young people and especially, but not exclusively, in the male homosexual context [78]. At the moment, there are no studies that investigate the prevalence of women addicted to ChemSex.

### Marijuana

Smokers and consumers of Marijuana and its derived, defined Hashish, because of their active agent (delta-9-tetrahydrocannabinol, THC) can incur in several risks, above all in cases of chronic abuse. In this regard, many studies investigated the psychological health and the possible exacerbation of psychotic diseases [79]. On the other hand, more controversial is the relationship between marijuana use and sexual functioning. A recent survey by Sun and Eisenberg [80] suggested, with also a certain clamor by traditional and social media, that marijuana consumers have a higher frequency of sexual intercourses compared to non-consumers. However, authors clearly declared the limitations of their study due to self-reported information from participants that limited the direct association between marijuana use and sexual frequency. Another article reports that marijuana and cannabinoids negatively impact and concurs to the physiological decline on testosterone levels in males, while in females marijuana is linked to major facilitatory effects [81].

### Cocaine

Cocaine use induces an increase in fantasies, physical sensitivity during excitement, ability to reach orgasm and sexual satisfaction in both sexes. However, these effects only occur in the early stages of consumption. Long-term cocaine use has a depressive effect on sexual activity [82], also leading to a reduction in the ability to reach orgasm [82]. The use of the substance, sometimes initiated to facilitate sexual stimulation, may cause alterations to the sexual sphere in the long term [83], provoking alterations in the endocrine system and influencing above all the hormonal balance controlling sexual function leading to the loss of sexual desire [83]. They also may produce impotence in man and sexual inactivity in the woman [84]. There is also inhibition of ejaculation [85] and a reduction in sexual performance [84].

### Amphetamine

Amphetamine is a stimulant of the sympathetic neurovegetative system. Its short-term use results in an increase in motor activity and sexual desire, a greater sense of power and self-esteem. Some consumers report that the use of amphetamine intensifies orgasm and allows prolonged sexual intercourse [86]. It is a stimulant to the sympathetic neurovegetative system and as such has been considered as a pharmacological aid in those affected by delayed ejaculation [86], a symptom characterized by absence of approved medical therapies [86].

In the long term, however, as all illegal drugs, the sexual symptoms appear, as a decrease in sexual desire, the inhibition of orgasm and, consequently, a reduction of sexual satisfaction [87].

### Heroin

The use of heroin initially proves physical well-being, a feeling of calm and serenity and it increases a feeling of self-confidence. It is often used to diminish the sense of sexual inadequacy, to combat performance anxiety, to improve sexual performance or to control ejaculatory reflex [77]. In women, heroin can be used to reduce the anxiety and pain associated with the relationship as it produces relaxation of the pelvic floor muscles and perhaps an increase in lubrication [77].

The toxic effects associated with prolonged and continuous use of heroin cause a rapid decrease in overall sexual helth. This is because heroin interferes with the central regulation of the endocrine system and therefore alters the functions involved in hormone processes interacting with the endogenous opioid receptors and pathway [88, 89].

Heroin-dependent men report several sexual complaints such as loss of libido, ED [90], delayed ejaculation, inability to reach orgasm and infertility [91].

### Ecstasy

In the early stages of assumption, ecstasy can be used in the attempt to overcome the shyness or discomfort associated with a sexual encounter. It gives a sense of mastery of self, of one’s own body and of its sexuality [92]. Some research has shown that the substance can lead to an increase in arousal or sensitivity, and in particular to the loss of sexual inhibition [92]. This phenomenon depends on methamphetamine, a powerful neurostimulator that can amplify the feeling of well-being and excitement and maximize sexual experience and orgasm [86]. Long-term ecstasy intake results in a reduction of sexual desire, penile erection, vaginal lubrication, ability to reach orgasm and being sexually satisfied [93].

## Chronic stress

Many people are daily exposed to experiences to small chronic stressors, which are physiological and lead to beneficial effect on health, motivation, performance, and emotional well-being (eustress) [94]. An accumulation of constant, small stressors, as important traumatic events (distress), can potentially contribute to sexual dysfunction [95]. Although stress is generally considered a major contributor of sexual dysfunction, there are only a few studies in the literature that investigate the effect of chronic stress on sexual function and more generally on the quality of life and satisfaction of the couple relationship [96, 97].

Chronic stress is generally defined as “an important life events that induce a prolonged period of stress such as a death in the family” [98] or as “a collection of small stressors that are constantly or frequently present, such as deadlines that never seem to be satisfied, traffic or financial concerns” [99, 100].

From a physiological perspective, an increase in chronic tension induces high levels of cortisol, which can cause harmful effects if it remains altered in the long term. For example, high levels of cortisol cause a reduction in gonadic steroids [101, 102] and adrenal androgens [103–107], which have been shown to have facilitating effects on sexual desire and genital arousal.

From a psychological point of view, stress can alter emotional and cognitive states, preventing the individual from focusing on sexual stimuli during sexual activity. The presence of chronic stress seems therefore to produce harmful effects on sexual desire and genital arousal [104, 108].

It is well known that there is a vicious circle in which sexual dysfunction, chronic stress, anxiety and depression compromise the quality of life of an individual [109]. In this context, it is necessary to accompany pharmacological therapy to the pathways of psychological counseling and psychotherapy [110–112].

In a prospective study of patients with ED, the group benefiting both PDE5i treatment and stress management techniques (diaphragmatic breathing exercises and progressive muscle relaxation) had lower levels of morning cortisol and better scores at IIEF compared to the group of patients who followed only pharmacological therapy. These findings highlighted how high levels of cortisol can inhibit sexual activity and develop sexual symptoms such as ED [113].

In another study, a sample of women with average to high stress levels was recruited and subjected to the viewing of an erotic movie. Women with high stress levels had lower levels of genital, but not psychological arousal, higher levels of cortisol, and reported more distraction during the erotic film than women in the average stress group [114]. Thus, high levels of chronic stress were related to lower levels of genital sexual arousal. Both psychological and hormonal factors were related to the lower levels of sexual arousal seen in women with high chronic stress levels [114].

## Conclusions

Sexual health, a crucial and pivotal part of overall health, is a complex interplay of cultural, social, relational, intrapsychic, and biomedical aspects [115, 116]. In presence of several unhealthy lifestyles, often sexual dysfunction occurs, which mostly represent the precursor of an underlying physical or mental health condition (See Table 1) [117].

**Table 1:** Risky and protective lifestyles and their effects on male and female sexuality

Factor	Effects on male sexuality	Effects on female sexuality
Obesity	Loss of libido [8] Risk of ED > 30% with BMI > 28.7 [13]	Arousal problems, Coital pain and Sexual dissatisfaction [8] More sexual complaints in women seeking bariatric surgery [22] Higher risk for postmenopausal breast cancer [27, 28]
Loss of weightand Diet	10% weight loss related with increased insulin sensitivity, plasma testosterone levels, erectile function and desire both in diabetics and non-diabetics [52] Weight loss induced by bariatric surgery improve erectile functions and testosterone levels [53] Mediterranean diet improves IIEF scores after 2-years [54]	Adherence to Mediterranean diet improve BMI, waist circumference, waist-to-hip ratio, mood symptoms, weight and metabolic syndrome [55] Higher frequency of sexual intercourses in women who better comply to Mediterranean diet [55]
Physical Activity	Protective effects against ED in men with diabetes [34] Increase of NO production and decrease of oxidative stress [14] Benefits on self-esteem and mental health [36, 41] Improvement of IIEF-5 score after 3 months of physical exercise [38] Augmented improvement of ED symptoms in association with PDE5i treatment [39] Sedentary life increase PE symptoms [41]	Protective effects against sexual dysfunction in women with diabetes [35] Benefits on self-esteem and mental health [36, 52] Improvement of menopause symptoms, mood, anxiety and musculoskeletal problems [47–49] Keeping good sexual quality of life in postmenopausal women who do regular physical activity [48] Regual physical exercises prevent the development of sexual dysfunction [51]
Smoking	Quit with smoking improve erectile function by 25% after 1 year [61] > 50% odds to develop ED in smokers and 30% in ex-smokers [60]	30% decrease of genital stimulation due to nicotine acute intake [62] Delayed orgasm and low vaginal lubrication [10, 64, 65]
Alcohol abuse	Lower likelihood of developing ED between non-habitual drinkers compared to binge-drinkers [68, 71]	Low sexual desire, inability to reach orgasm or unsatisfactory orgasm in women with an alcohol dependence [68, 73]
Marijuana	Physiological decline of testosterone levels [81]	It gives major facilitatory effects [81]
Cocaine	Depressive effect on sexual activity and progressive inability to reach orgasm in the long term [82, 83] Loss of sexual desire, ED symptoms, inhibition of ejaculation and reduction in sexual performance [83, 85]	Loss of sexual activity [84]
Amphetamine	Decrease in sexual desire, inhibition of orgasm and a reduction of sexual satisfaction [87]	
Heroin	At early stages, it improves sexual inadequacy, sexual performance and ejaculatory control [88] Heroin dependence produces loss of libido and ED [90] Heroin dependence produces delayed ejaculation or inability to reach it [91]	At early stages, it reduces anxiety and coital pain, due to a relaxation of pelvic floor muscles and higher lubrication [88]
Ecstasy	At early stages, it reduces sexual inhibition [92] Long-term use reduces sexual desire and penile erection [93]	At early stages, it maximized sexual experience and orgasm [77] Long-term use reduces vaginal lubrication and ability to reach satisfying orgasm [93]
Chronic stress	PDE5i treatment combined with stress management techniques reduces cortisol levels and improves IIEF scores [113]	Chronic stress is related to lower levels of sexual arousal both in hormonal factors (cortisol) and psychological factors (attention to visual sexual stimuli) [114]

The analysis of the literature about the impact of healthy and unhealthy lifestyles on sexuality reported differences in men and women but also similar effects. For example, alcohol abuse seems to produce different negative effects in men and women: reduction of erectile function in the first case, paradox loss of libido and unsatisfactory orgasms in the second one; inversely, physical activity increase sexual function and prevent sexual dysfunctions in both middle age men and women. A better comprehension of these gender differences should be more deeply studied in future researches.

Independently from the organic or non-organic cause of sexual dysfunction [118], it is important to note that the promotion of correct lifestyles represent the first-line therapeutic option for both sexual physicians and psychosexologists. Changes in lifestyle like alcohol, smoking, and illegal drug cessation, control of diabetes / dyslipidemia, (re)starting physical activity, weight loss and reduction in stress are of prime importance [119].

Finally, in evaluating and treating subjects with sexual dysfunctions, it is important to take care of patients on a systems (previously called biopsychosocial) perspective [1] and recognize that both physical factors (obesity, smoking, alcohol and substance abuse) and psychosocial factors (stress, anxiety, depression, income, culture, experiences, and personality) can not only derive from the sexual dysfunction but they also may contribute to and amplify it. On the other hand, combining efficacious pharmacotherapies with successful additional modalities (such as sexual counselling, sexual education or psychotherapy) to reduce sexual symptoms, is to be regarded as a unique and strong tool in order to motivate the needed, but difficult and hard to perform lifestyle modifications requested to the patient [109].
